# Detection of Participation and Training Task Difficulty Applied to the Multi-Sensor Systems of Rehabilitation Robots

**DOI:** 10.3390/s19214681

**Published:** 2019-10-28

**Authors:** Hao Yan, Hongbo Wang, Luige Vladareanu, Musong Lin, Victor Vladareanu, Yungui Li

**Affiliations:** 1 Parallel Robot and Mechatronic System Laboratory of Hebei Province, Yanshan University, Qinhuangdao 066004, China; yh@stumail.ysu.edu.cn (H.Y.); lms19910704@163.com (M.L.); lyg@stumail.ysu.edu.cn (Y.L.); 2 Academy for Engineering & Technology, Fudan University, Shanghai 200433, China; 3 Robotics and Mechatronics Department, Institute of Solid Mechanics of Romanian Academy, 010141 Bucharest, Romania; victor.vladareanu@imsar.ro

**Keywords:** rehabilitation robot, human–robot interaction, training task, multi-sensor system, quantum particle swarm optimization, support vector machines

## Abstract

In the process of rehabilitation training for stroke patients, the rehabilitation effect is positively affected by how much physical activity the patients take part in. Most of the signals used to measure the patients’ participation are EMG signals or oxygen consumption, which increase the cost and the complexity of the robotic device. In this work, we design a multi-sensor system robot with torque and six-dimensional force sensors to gauge the patients’ participation in training. By establishing the static equation of the mechanical leg, the man–machine interaction force of the patient can be accurately extracted. Using the impedance model, the auxiliary force training mode is established, and the difficulty of the target task is changed by adjusting the K value of auxiliary force. Participation models with three intensities were developed offline using support vector machines, for which the C and σ parameters are optimized by the hybrid quantum particle swarm optimization and support vector machines (Hybrid QPSO-SVM) algorithm. An experimental statistical analysis was conducted on ten volunteers’ motion representation in different training tasks, which are divided into three stages: over-challenge, challenge, less challenge, by choosing characteristic quantities with significant differences among the various difficulty task stages, as a training set for the support vector machines (SVM). Experimental results from 12 volunteers, with tasks conducted on the lower limb rehabilitation robot LLR-II show that the rehabilitation robot can accurately predict patient participation and training task difficulty. The prediction accuracy reflects the superiority of the Hybrid QPSO-SVM algorithm.

## 1. Introduction

Neuromuscular injury can lead to disability or inconvenient movements, such as stroke and spinal cord injury, which have become important problems in the world [1]. Nowadays, there are more than 33 million stroke patients in the world [2], the mortality rate is as high as 80%, and 75% of the survivors are disabled [3]. The necessity to develop rehabilitation robots has made it one of the research hotspots in the world [4,5]. As a robot that is in direct contact with the patient, the rehabilitation robot shoulders the responsibility of helping the patient recover smoothly and safely. The human–computer interaction strategy, the energy interaction and role distribution control, are very important [6].

Clear detection of human–computer interaction and patient intention are the basis of flexible robot control. Most rehabilitation robots use force sensors to feedback mechanical information from patients, such as Hongbing Tao [7], Victor G [8]. Hwang et al. judged human motion intention by collecting pressure sensor data placed at the contact point between the standing posture rehabilitation robot and stroke patients [9]. Wolf S et al. connected the elastic element in series with the driving part and named it the Serial elastic actuator. By detecting the deformation of the elastic element, the joint moment can be detected and the motion intention of the patient can be judged [10]. Kim et al. used only one pressure sensor to realize the assistant force of the robot in the motion of the patient’s elbow joint [11]. Some of them rely on current changes of the joint motors to detect motion intentions, such as Kim [12]. A few researchers use surface EMG (electromyography) signals and EEG (electroencephalogram) signals to predict the patients’ motor intentions [13,14], such as Edward [15], Magdalena [16], and Tang [17]. Yepes et al. use electromyogram signals to determine the required moment of the knee joint [18]. Some researchers apply EMG signals in motion modal recognition of the prosthesis [19] and Cooperative Robot [20]. All these measurement methods have their own advantages, but EEG is susceptible to noise interference [21]. The EMG signal is not easy to collect when the skin surface changes [22,23]. Relatively speaking, it does not increase the cost of the robot and the complexity of the system. In this paper, the joint torque sensor and the hardware detection system of the six-dimensional force sensor on the sole are used.

Meanwhile, the rehabilitation effect is not only related to scientific rehabilitation training methods and reasonable training planning, but also has a great impact on the patients’ active participation and active sports intention, which has been proven by clinical studies [24]. In order to improve the active participation of patients during the training process, it is necessary to provide assistance to patients according to the interaction situation during the training process [25] and to maximize the patient’s independent tasks. Introducing patients’ movement characteristics and physical fitness into the control strategy has a positive impact on the rehabilitation effect on the patients [26]. Yatsenko et al. controlled and adjusted the movement speed of the robotic arm according to the amplitude ratio of the EMG signal of the affected limb [27], and the patient could quickly adapt to control the movement of the prosthesis [28], but it was inconsistent with the movement characteristics of the human body. Many researches have introduced velocity field and virtual channel technology in the specific trajectory of rehabilitation training [29,30]. Cai uses impedance control to construct the velocity field in different directions of expected trajectories and provide correction force within a certain range of trajectories, with the correction force being a rigid force outside the interval threshold [31], but the threshold size setting is not given. In order to provide more accurate training for patients, radial basis function (RBF) neural network has excellent analytical ability in the patient’s motor ability analysis, which was researched by Wolbrecht [32] and Pehlivan [33]. However, during the training period, the rehabilitation robot frequently interferes with the training of patients, which easily causes the side effect of relying on the machine, and fails to motivate the active participation of patients. In order to introduce the training state of patients into the control loop more accurately, many studies have judged the patient’s psychological-level participation by collecting the patient’s EMG signal, EEG signal, and other physiological information [34,35,36]. At the same time, in order to stimulate patients to actively participate in training, most rehabilitation robots currently use interesting games [37] or virtual reality technology [38].

In summary, this paper proposes a lower limb rehabilitation robot using joint torque sensors and six-dimensional force sensors on the foot soles. In the training task, man–machine interaction force information is collected, from which can be extracted characteristic quantities to predict the task difficulty by using support vector machines. The rest of this paper is organized as follows: the second section introduces the rehabilitation robot structure of multi-sensor system and human–machine interaction mechanical model. In the third section, a multi-difficulty rehabilitation training task is proposed. Under the model of impedance control, a support vector machines algorithm is used to establish the model of the patients’ active participation and task difficulty detection. The fourth section analyses the characteristic quantity of 10 healthy volunteers during different difficulty training tasks, using support vector machines (SVM) to predict the participation and task difficulty of two other volunteers.

## 2. LLR-II Rehabilitation Robot

### 2.1. Structural Design of LLR-II

**Figure 1:**
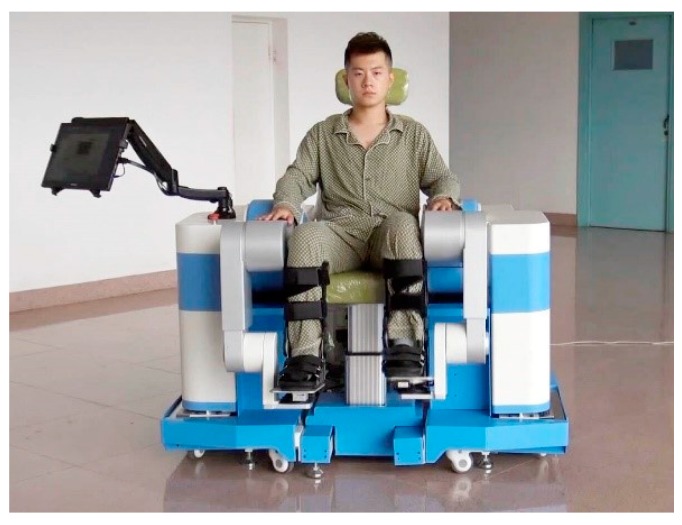
The LLR-II Rehabilitation.

In order to adapt to the patients in the early stage of rehabilitation, the lower limb rehabilitation robot LLR-II designed in this paper can be trained in two postures, so as to prevent the mechanical leg from squeezing the patient [39,40]. As to the hardware platform of the rehabilitation system, LLR-II adopts a modular design and consists of five sub-modules: lower limb mechanical leg, main control system, sensor system, multi-function seat, and mechanical limit adjustment frame, as shown in Figure 1, to validate that the rehabilitation robot can accurately predict subjects participation and training task difficulty.

**Figure 2:**
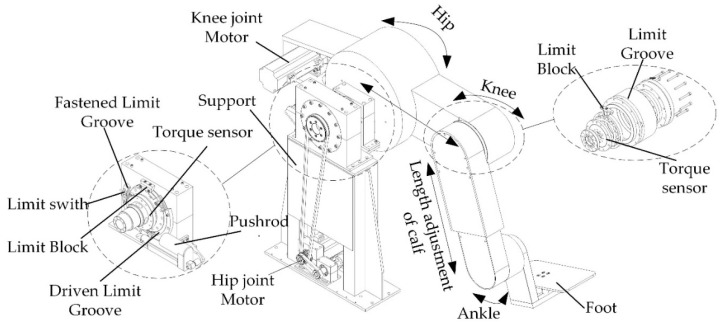
The detailed design of LLR-II leg mechanism.

The mechanical leg is a planar three-degree-of-freedom serial mechanism, similar to the three joints of human leg, including hip, knee, and ankle. In order to solve the problem of excessive driving power of the hip joint, a self-balancing design is adopted. The knee drive component is installed on the back of the hip joint rotation axis to balance the weight of part of the mechanical leg, reduce the driving power of the hip joint, and improve the dynamic performance of the mechanical leg. The addition of an electric pushrod in the mechanical leg can automatically adapt to patients with a height of 1500 mm to 1900 mm. In order to realize the safety of sitting and lying posture training for patients, variable joint limitation consisting of fastened limit groove and driven limit groove was designed, as shown in Figure 2.

**Figure 3:**
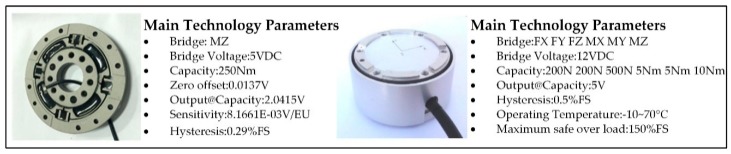
The detailed design of LLR-II leg mechanism.

Torque sensors are mounted inside the hip and knee joint of the LLR-II sagittal plane to detect the dynamic torque characteristics of the patient’s training state in real time. The dynamic torque characteristic of the ankle joint is detected by a six-dimensional force sensor mounted on the sole of the foot. The torque sensor is manufactured by Sunrise Instruments Company in China, and the six-dimensional force sensor is manufactured by Junde Technology Co., Ltd. in China. The profile of the sensor and the sensor’s detailed parameters are shown in Figure 3. The output side of the reducer increases the sensitivity and accuracy of the mechanical information detection and uses this information to complete the patient’s motion intention detection.

**Figure 4:**
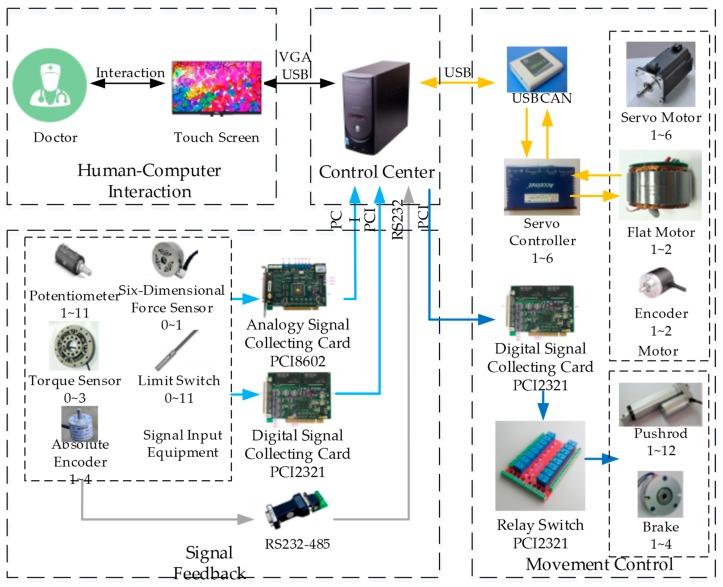
The sensors system composition of LLR-II.

Based on the LLR-II rehabilitation training function, its electrical control system is divided into Control Center system, Movement Control system, Signal Feedback system, Human–Computer Interaction system, as shown in Figure 4. The control center system use a variety of sensors to monitor the human–computer interaction state, and uses a variety of signals to complete the planning and training tasks; the robotic arm receives the instructions and drives the affected limbs to perform the multi-mode advanced rehabilitation training under the guidance of the driving system.

### 2.2. Man–Machine Interaction Mechanics Model of LLR-II

The joint no-load moment in LLR-II man–machine coupled motion is affected by the weight of the mechanical leg and the patient’s leg, and it can be expressed as a nonlinear function of joint variables [41].

(1) $$M_{n\times 1}=F(\theta_{n\times 1})$$

where, $M_{n\times 1}$ is the column vector of joint no-load torque, F(•) is the mapping function, and $\theta_{n\times 1}$ is the joint variable.

**Figure 5:**
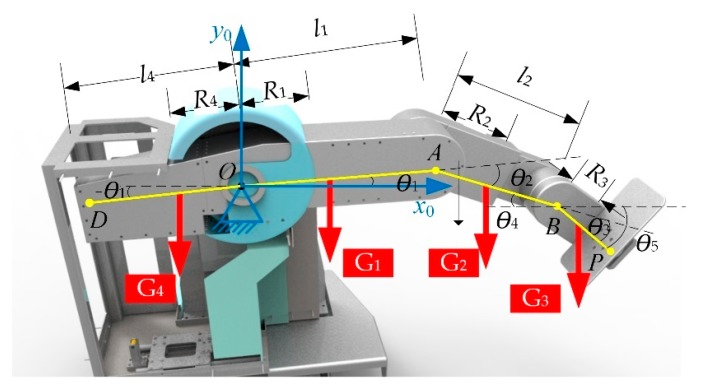
Leg model of lower limb rehabilitation robot.

According to its own structure, it can be simplified as a planar three-link series mechanism. It should be noted that, considering the large weight of the leg, in order to increase the stability of the hip joint, the self-balancing design concept was introduced in the design process. The specific model can be shown in Figure 5.

In the Figure, $l_{1}–l_{3}$ represent the length of the thigh, calf, and sole, respectively; $l_{4}$ represents the length of the self-balancing part; O,A,B represent the hip, knee, and ankle joints, respectively; D,P respectively represent the first and last two endpoints; $G_{1},G_{2},G_{3}$ represent the weight of the machine and the patient’s thigh, calf, and foot, respectively; $G_{4}$ represents the weight of the self-balancing part; $R_{1}–R_{4}$ represent the lengths from the center of gravity of each part to the node; $\theta_{1},\theta_{2},\theta_{3}$ represent the joint variables, in a counter-clockwise positive direction; $\theta_{4},\theta_{5}$ represent the intermediate quantity introduced.

The joint no-load moment equation is obtained as

(2) $$\left[\begin{array}{l} M_{1}\\ M_{2}\\ M_{3} \end{array}\right]=\left[\begin{array}{ccc} \cos\theta_{1} & \cos\theta_{4} & \cos\theta_{5}\\ 0 & \cos\theta_{4} & \cos\theta_{5}\\ 0 & 0 & \cos\theta_{5} \end{array}\right]\left[\begin{array}{l} G_{3}l_{1}+G_{2}l_{1}+G_{1}R_{1}-G_{4}R_{4}\\ G_{3}l_{2}+G_{2}R_{2}\\ G_{3}R_{3} \end{array}\right]$$

In combination with Equation (2), the above equation can be modified to:

(3) $$\left[\begin{array}{l} M_{1}\\ M_{2}\\ M_{3} \end{array}\right]=\left[\begin{array}{ccc} \cos\theta_{1} & \cos(\theta_{1}+\theta_{2}+\theta_{3}) & \cos(-\theta_{2}-\theta_{1})\\ 0 & \cos(\theta_{1}+\theta_{2}+\theta_{3}) & \cos(-\theta_{2}-\theta_{1})\\ 0 & 0 & \cos(-\theta_{2}-\theta_{1}) \end{array}\right]\left[\begin{array}{l} G_{3}l_{1}+G_{2}l_{1}+G_{1}R_{1}-G_{4}R_{4}+f_{1}\\ G_{3}l_{2}+G_{2}R_{2}+f_{2}\\ G_{3}R_{3}+f_{3} \end{array}\right]$$

It can be abbreviated as:

(4) $$M_{3\times 1}=L_{3\times 3}(\theta )•C_{3\times 1}$$

In the formula, the joint no-load torque term is represented by $M_{3\times 1}$, and the joint variable term is $L_{3\times 3}(\theta )$, and $C_{3\times 1}$ is a characteristic parameter term.

The characteristic parameter item $C_{3\times 1}$ is associated with patient information, which is unique to any patient, and needs to be solved for each patient. Since joint variables $L_{i}$ and $\theta_{i}$ can be measured by the sensor system on the robot body, but $G_{i}$ cannot be directly measured by the weight of the patient’s leg, the measured torque $M_{s}$ is the sum of applied torque $M_{h}$ and no-load torque M:

(5) $$M_{s}=M_{h}+M$$

And can be obtained from Equation (4) as,

(6) $$C_{3\times 1}=L_{3\times 3}{\left(\theta \right)}^{-1}•M_{3\times 1}$$

First, the ankle joint is moved at a small speed V, the foot pressure value $f_{z}{}_{d}$ at this time is recorded at intervals Δt, the joint angles *θ*_1_, $\theta_{2}$, and $\theta_{3}$ are calculated, and a total of k times are recorded. Then the knee joint is rotated k times in the same manner, knee joint torque value $M_{2}$ and angle values $\theta_{1}$,$\theta_{2}$ and $\theta_{3}$ are recorded, then the hip joint is rotated to record k hip joint torque values $M_{1}$ and angle values $\theta_{1}$,$\theta_{2}$ and $\theta_{3}$, then $f_{z}{}_{d}$, $\theta_{3}$,$\theta_{2}$, $\theta_{1}$ are converted into $M_{1}$,$\theta_{4}$,$\theta_{5}$. $C_{31}$, $C_{21}$ and $C_{11}$ are calculated according to the following formulae.

(7) $$\begin{array}{l} M_{3}{}_{i}=\cos\theta_{5}{}_{i}C_{31i}\;(i=1-k)\\ {\bar{C}}_{31}=\frac{1}{k}\sum_{i=1}^{k}\frac{M_{3}{}_{i}}{\cos\theta_{5}{}_{i}} \end{array}$$

(8) $$\begin{array}{l} M_{2}{}_{i}=\cos\theta_{4}{}_{i}C_{21i}+\cos\theta_{5}{}_{i}{\bar{C}}_{31}\;(i=1-k)\\ {\bar{C}}_{21}=\frac{1}{k}\sum_{i=1}^{k}\frac{M_{2}{}_{i}-\cos\theta_{5}{}_{i}{\bar{C}}_{31}}{\cos\theta_{4}{}_{i}} \end{array}$$

(9) $$\begin{array}{l} M_{1}{}_{i}=\cos\theta_{1}{}_{i}C_{11i}+\cos\theta_{4}{}_{i}{\bar{C}}_{21}+\cos\theta_{5}{}_{i}{\bar{C}}_{31}\;(i=1-k)\\ {\bar{C}}_{11}=\frac{1}{k}\sum_{i=1}^{k}\frac{M_{1}{}_{i}-\cos\theta_{5}{}_{i}{\bar{C}}_{31}-\cos\theta_{4}{}_{i}{\bar{C}}_{21}}{\cos\theta_{4}{}_{i}} \end{array}$$

(10) $$C_{3\times 1}={\left[\begin{array}{ccc} {\bar{C}}_{11} & {\bar{C}}_{21} & {\bar{C}}_{31} \end{array}\right]}^{T}$$

The force exerted by the patient’s active intention is the main feature to be identified in rehabilitation training. We judge the rehabilitation effect of the patient by identifying the force that the patient can produce actively. In the training process, the actual measurement of human–machine interaction force is the data measured by the sensor system of the robot. The following equation is the established equivalent terminal mechanical model of human patients.

(11) $$f_{2\times 1}=H(\theta_{3\times 1},M_{3\times 1},M_{s}{}_{3\times 1})$$

In the formula, $f_{2\times 1}$ is static terminal forces in the plane of motion, $\theta_{3\times 1}$ is current position lower joint variable, $M_{3\times 1}$ is three-joint no-load torque, $M_{s}{}_{3\times 1}$ is measured force/torque at the three joints, and H(•) is the mapping function.

**Figure 6:**
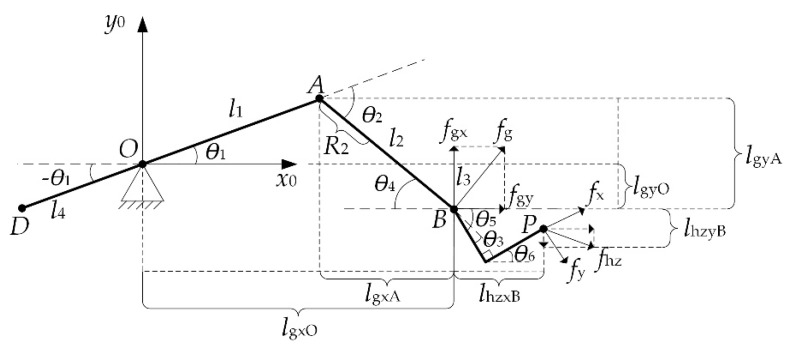
End applied force model.

In the process of human–machine motion, since the force exerted by the patient mainly acts on the pedal, a six-dimensional force sensor is placed in the middle of the bottom of the pedal. Due to the influence of the arch structure of the foot, the force at the heel and the forepaw is simplified to two points: B and P. The force at the heel generates torque mainly at the hips and knees, and the force at the forepaw generates pressure on the foot pedal, as shown in Figure 6.

In Figure 6, $f_{g}$ represents the patient applying force at point B; ***f_gx_***, $f_{g}{}_{y}$ and denote the horizontal and vertical resolution of $f_{g}$, respectively; $f_{hzx},f_{hzy}$ represent the horizontal and vertical resolution of foot forepaw forces, respectively; $l_{gxO},l_{gxA}$ represent the moment arms generated by $f_{g}{}_{x}$ at point A and O, respectively; $l_{gyO},l_{gyA}$ represent the moment arm generated by $f_{g}{}_{y}$ at point A and O, respectively; $l_{hzxB}$, $l_{hzyB}$ represent the moment arms generated by $f_{hzx}$ and $f_{hzy}$ at point **B**; $f_{x}$ and $f_{y}$ represent the force measured by the sensor mounted on the sole of the robot foot, respectively.

The moment of hip and knee joint can be expressed as:

(12) $$\left[\begin{array}{c} M_{h1}\\ M_{h2} \end{array}\right]=\left[\begin{array}{cc} l_{2}\sin\theta_{5}+l_{1}\sin\theta_{1} & l_{2}\cos\theta_{5}+l_{1}\cos\theta_{1}\\ l_{2}\sin\theta_{5} & l_{2}\cos\theta_{5} \end{array}\right]\left[\begin{array}{c} f_{gx}\\ f_{gy} \end{array}\right]$$

The patient’s heel force $f_{g}$ can be expressed as:

(13) $$f_{g}=\left[\begin{array}{c} f_{gx}\\ f_{gy} \end{array}\right]={\left[\begin{array}{cc} l_{2}\sin\theta_{5}+l_{1}\sin\theta_{1} & l_{2}\cos\theta_{5}+l_{1}\cos\theta_{1}\\ l_{2}\sin\theta_{5} & l_{2}\cos\theta_{5} \end{array}\right]}^{-1}\left[\begin{array}{c} M_{h}{}_{1}\\ M_{h}{}_{2} \end{array}\right]$$

From which can be obtained

(14) $$f_{g}=\frac{\left[\begin{array}{cc} l_{2}\cos\theta_{5} & -(l_{2}\cos\theta_{5}+l_{1}\cos\theta_{1})\\ -l_{2}\sin\theta_{5} & l_{2}\sin\theta_{5}+l_{1}\sin\theta_{1} \end{array}\right]\left[\begin{array}{c} M_{s}{}_{1}-M_{1}\\ M_{s}{}_{2}-M_{2} \end{array}\right]}{\left(\left(l_{2}\sin\theta_{5}+l_{1}\sin\theta_{1}\right)(l_{2}\cos\theta_{5})-(l_{2}\sin\theta_{5})(l_{2}\cos\theta_{5}+l_{1}\cos\theta_{1})\right)}$$

The force exerted on the patient’s forefoot is collected by a six-dimensional force sensor on the sole of the foot that has the same axial direction as the pedal, so the end force $f_{hz}$ can be decomposed as follows:

(15) $$f_{hz}=\left[\begin{array}{c} f_{hz}{}_{x}\\ f_{hz}{}_{y} \end{array}\right]=\left[\begin{array}{c} f_{x}\cos\theta_{6}+f_{y}\sin\theta_{6}\\ f_{x}\sin\theta_{6}-f_{y}\cos\theta_{6} \end{array}\right]$$

Then, the equivalent terminal force of the patient can be calculated as:

(16) $$f=f_{g}+f_{hz}=\left[\begin{array}{c} F_{x}\\ F_{y} \end{array}\right]$$

where $F_{x}$, $F_{y}$ are the horizontal and vertical components of terminal force;

By combining Equations (13) and (15), the terminal static component can be expressed as follows:

(17) $$\begin{array}{l} F_{x}=\frac{l_{2}\cos\hat{\theta }(M_{s}{}_{1}-M_{1})-(l_{2}\cos\hat{\theta }+l_{1}\cos\theta_{1})(M_{s}{}_{2}-M_{2})}{\left(\left(l_{2}\sin\hat{\theta }+l_{1}\sin\theta_{1}\right)(l_{2}\cos\hat{\theta })-(l_{2}\sin\hat{\theta })(l_{2}\cos\hat{\theta }+l_{1}\cos\theta_{1})\right)}+\\ \;f_{x}\cos\left(\theta_{1}+\theta_{2}+\theta_{3}+\pi /2\right)+f_{y}\sin\left(\theta_{1}+\theta_{2}+\theta_{3}+\pi /2\right) \end{array}$$

In the formula, $\hat{\theta }$ is intermediate variable of joint Angle, $\hat{\theta }=-\theta_{1}-\theta_{2}$.

(18) $$\begin{array}{l} F_{y}=\frac{-l_{2}\sin\hat{\theta }(M_{s}{}_{1}-M_{1})+(l_{2}\sin\hat{\theta }+l_{1}\sin\theta_{1})(M_{s}{}_{2}-M_{2})}{\left(\left(l_{2}\sin\hat{\theta }+l_{1}\sin\theta_{1}\right)(l_{2}\cos\hat{\theta })-(l_{2}\sin\hat{\theta })(l_{2}\cos\hat{\theta }+l_{1}\cos\theta_{1})\right)}+\\ \;f_{x}\sin\left(\theta_{1}+\theta_{2}+\theta_{3}+\pi /2\right)-f_{y}\cos\left(\theta_{1}+\theta_{2}+\theta_{3}+\pi /2\right) \end{array}$$

Equations (17) and (18) can completely solve the mapping relationship between terminal force and joint variables, no-load torque and measured torque mentioned in Equation (11), and provide the entry parameters for the following judgment of patients’ motion intention and control strategy.

## 3. Participation Detection of LLR-II

### 3.1. Assist Force Training Control

According to the change of the patient’s participation in the training process, the assist mode and the active training mode are divided into different grades to ensure that the patient completes the training and maximize the patient’s training enthusiasm and task completion. Using the impedance control model, the human–computer interaction force is represented by the end position offset, and is magnified by game in the task. With the participation of the robot’s assistant force, the task difficulty is classified, to ensure that patients can find suitably challenging rehabilitation tasks. In order to improve the level of the patients’ active participation, according to the recognized level of physical participation, the size of the auxiliary force is adjusted in real time to ensure that patients maintain a high level of participation for training.

**Figure 7:**
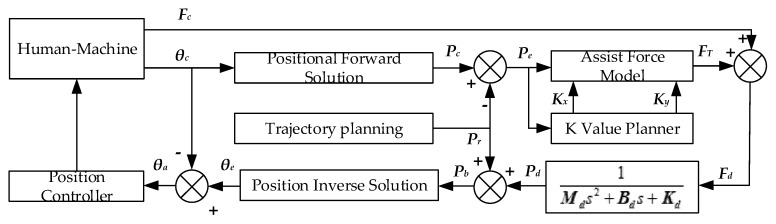
Assistance training control block diagram.

With the progress of rehabilitation training, the patient gradually has a certain ability to control the affected limb, but when it is not enough to fully control it, it is necessary to introduce assistance training, in which the robot obtains the patient’s motion intention through the force/torque sensor, and then drives the affected limb for training. In order to improve the coordination ability of each joint, the patient needs to complete the trajectory training, such as the circular trajectory and the linear trajectory. In many cases, the patient does not have the ability to perform the trajectory training task independently, and the robot needs to assist in suppressing the wrong movement. The assistance training control mode introduces the impedance model as shown in Figure 7. According to the current joint actual position $\theta_{a}$, the position positive solution is compared with the current desired position of the training trajectory, the auxiliary force calculation is performed, the auxiliary force $F_{T}$ is obtained, the patient force $F_{a}$ is summed, and the result is sent to the impedance controller to obtain the end position control amount $P_{d}$. It then inversely solves the position, calculates the desired joint position $\theta_{d}$, and transmits it to the position controller to realize the assist control.

In order to make patients intuitively understand the movement track of their affected limbs, a task game was designed in which the patient operated the virtual mice to walk in the safe area between the red lines. The position of the mouse on the screen reflects the position of the patient’s limb end in the motion plane. Participation scores increased with the time the mice spent walking in the safe area, and did not increase when the mice were outside the safe area. The trajectory of the safe passage can be selected according to the length of the patient’s limb, and the width of the safe passage is related to the parameters of the impedance model. The impedance model parameters are as follows:

(19) $$M=\left[\begin{array}{c} 0.0625\\ 0.0625 \end{array}\right],B=\left[\begin{array}{c} 5\\ 5 \end{array}\right],K=\left[\begin{array}{c} 1000\\ 1000 \end{array}\right]$$

In order to make the width of the safety passage between the red lines challenging for most patients, and not tedious at the same time, 70 kg is selected as the standard reference value for the weight of patient, and the positional offset of the standard reference value is selected as the width of the safety channel. The standard reference value of 70 kg was selected as appropriate to the lot of volunteers that the machine was tested on, and it is used to provide an approximate starting point to the initial conditions of the algorithm. During the training task, patients need to resist the weight of their limbs and control the virtual mice to walk at a constant speed in the safe passage. If the virtual mice touch the red line during the task, the physical strength of the mice will decline until the end of the training mission cycle. According to the size of the auxiliary force, the task difficulty is divided into nine levels, with K values ranging from 0 to 0.8, respectively. The degree to which patients participate in training is related to the degree of the patient’s recovery and individual physical strength. These parameters are difficult to quantify. Therefore, the degree of the patients’ participation in training is quantified in stages by means of an experimental questionnaire. In the course of the experiment, patients are asked to try nine different difficulty training tasks, and they are asked to accept questionnaires to determine the current situation. Task difficulty is appropriate for each patient. Tasks with different difficulty can be divided into three states: under-challenge, challenge, and over-challenge, which are expressed by −1, 0, 1.

### 3.2. Patient Participation and Training Task Difficulty Prediction Model

In order to predict the degree of the patient’s task participation, a mathematical model based on support vector classifiers and regression machines was established according to the characteristics of the small sample and nonlinear data of a small number of patients‘ training data and questionnaire data. The characteristic parameters were extracted from the training data, and the data was analyzed. The implicit mathematical relationship between input value and output value predicts the actual participation of patients, so as to achieve the goal of selecting the appropriate task difficulty.

Using the characteristic quantity of patient training data and task difficulty states, a QPSO-MLSSVM (quantum particle swarm optimization and multi-output least squares support vector machine) model can be established and tested. This model is based on the LS-SVM (least squares support vector machines) model, which is a class of kernel-based learning methods normally used for regression and classification problems. The main distinction in LS-SVM is solving for a set of linear equations, rather than the quadratic programming problem in classical SVMs [42]. The QPSO (quantum particle swarm optimization) algorithm is used to optimize the key parameters in the model to make the model performance better [43,44]. The sample set is $\left\{\left(x_{i},y_{i}\right),i=1,2,\ldots ,l\right\}$, where $x_{i}\in R^{n}$ is the input value of the *ith* sample, $y_{i}\in R$ is the output value of the *nth* sample. The assumption is

(20) $$f_{i}\left(x\right)=\omega^{T}•\Phi \left(x_{i}\right)+b_{i},i=1,2,\ldots ,l$$

where, $\Phi \left(x_{i}\right)$ is the spatial conversion variable function, ω is the weight vector, b is the adjustment parameter.

We optimize the confidence interval under this condition, and transform the optimization problem into the minimum value problem according to the principle of structural risk minimization [45]:

(21) $$\{\begin{array}{l} \min\frac{1}{2}{\left\|\omega \right\|}^{2}+C\sum_{i=1}^{l}\xi_{i}\\ \begin{array}{l} s.t.y_{i}•f_{i}\left(x\right)\geq 1-\xi_{i},i=1,2,\ldots ,l\\ \begin{array}{ccc} & & \xi_{i}\geq 0,i=1,2,\ldots ,l \end{array} \end{array} \end{array}$$

where, C is the weight coefficient; $\xi_{i}$ is the relaxation factor.

The first item in the optimization problem reflects the generalization ability and the model complexity, the second item reflects the model error, and the parameter C adjusts the weight of these two items. Introducing the Lagrange equation into the above formula:

(22) $$\begin{array}{c} L=\frac{1}{2}{\left\|w\right\|}^{2}+C\sum_{i=1}^{l}\left(\xi_{i}+\xi_{i}^{*}\right)-\sum_{i=1}^{l}a_{i}\left(\varepsilon +\xi_{i}-y_{i}+\left(\omega^{T}•\Phi \left(x_{i}\right)+b\right)\right)\\ -\sum_{i=1}^{l}a_{{}_{i}}^{*}\left(\varepsilon +\xi_{{}_{i}}^{*}+y_{i}-\left(\omega^{T}•\Phi \left(x_{i}\right)+b\right)\right)-\sum_{i=1}^{l}\left(\eta_{i}\xi_{i}+\eta_{{}_{i}}^{*}\xi_{{}_{i}}^{*}\right) \end{array}$$

where $\xi^{\left(*\right)}$, $\alpha^{\left(*\right)}$ and $\eta^{\left(*\right)}$ represent ξ, α, η with * and without *, $\alpha^{\left(*\right)}$ and $\eta^{\left(*\right)}$ are Lagrange multipliers. A relaxation variable is introduced $\xi_{i,}\xi_{i}^{*}\geq 0,i=1,2,\ldots ,l$, ξ is the insensitive.

The radial basis function is selected to calculate the spatial inner product of the kernel function in the support vector machine model. The result obtained by the above formula is inserted back into the Lagrange equation to obtain the dual equation of the optimization function:

(23) $$\max W\left(a_{i},a_{i}^{*}\right)=-\frac{1}{2}\sum_{i=1}^{l}\sum_{j=1}^{l}\left(a_{i}-a_{i}^{*}\right)\left(a_{j}-a_{j}^{*}\right)\times K\left(x_{i},x_{j}\right)+\sum_{i=1}^{l}\left(a_{i}-a_{i}^{*}\right)y_{i}-\sum_{i=1}^{m}\left(a_{i}+a_{i}^{*}\right)\varepsilon_{i}$$

The constraint of this dual equation is:

(24) $$\{\begin{array}{c} \sum_{i=1}^{l}\left(a_{i}-a_{i}^{*}\right)=0\\ a_{i},a_{{}_{i}}^{*}\in \left(0,C\right) \end{array}$$

where, C>0 is the Penalty parameter

When $0<\alpha_{i}<C$, $\xi_{i}=0$; when $0<\alpha_{i}^{*}<C$, $\xi_{{}_{i}}^{*}=0$, the corresponding sample is the standard support vector, and expresses the reliability of the calculation. In general, the b value of the standard support vector is calculated respectively, and then the average value is calculated.

(25) $$\begin{array}{c} b=\frac{1}{N_{NSV}}\{\sum_{0<\alpha_{i}<C}\left[y_{i}-\sum_{x_{i}\in SV}\left(\alpha_{i}-\alpha_{i}^{*}\right)\Phi \left(x_{i}\right)•\Phi \left(x_{i}\right)-\varepsilon \right]\\ +\sum_{0<\alpha_{i}^{*}<C}\left[y_{i}-\sum_{x_{i}\in SV}\left(\alpha_{i}-\alpha_{i}^{*}\right)\Phi \left(x_{i}\right)•\Phi \left(x_{i}\right)+\varepsilon \right]\} \end{array}$$

In order to eliminate local optima problems, the QPSO algorithm and the SVM algorithm are mixed the Hybrid QPSO-SVM algorithm. The formula of QPSO is:

(26) $$\{\begin{array}{l} m_{best}=\frac{1}{M}\sum_{i=1}^{M}P_{i}\\ P_{C_{ij}}=\phi P_{ij}+\left(1-\phi \right)P_{gj}\\ x_{ij}\left(t+1\right)=P_{C_{ij}}\pm \alpha \left|m_{bestj}-x_{ij}\left(t\right)\right|\ln\left(\frac{1}{u}\right) \end{array}$$

And the particle swarm velocity formula is:

(27) $$v_{ij}\left(t+1\right)=\omega •v_{ij}\left(t\right)+c_{1}r_{1j}\left[P_{ij}\left(t\right)-x_{ij}\left(t\right)\right]+c_{2}r_{2j}\left[P_{gj}\left(t\right)-x_{gj}\left(t\right)\right]$$

where, $p_{ij}$, $p_{gj}$, $p_{ij}$ are the optimal positions of the i particle and the g particle in the j dimension, respectively; $m_{best}$, $m_{bestj}$ are center points of the current best position of all individuals on all dimensions; M is the particle swarm size; $p_{i}$ is the current best position of the i particle; $p_{c_{ij}}$ is the random position between $p_{ij}$, $p_{gj}$; α is the control coefficient.

QPSO optimizes two key parameters, C and σ of the MLSSVM, and the optimization goal is minimizing the fitness(σ,γ) function. The sample mean square error (MSE) is selected as the particle swarm fitness function.

(28) $$fitness\left(\sigma ,\gamma \right)=\frac{1}{M}{\sum_{i=1}^{M}\left(y_{i}-{\hat{y}}_{i}\right)}^{2}$$

where, $y_{i}$ is the actual value and ${\hat{y}}_{i}$ is the predicted value. When fitness reaches its minimum, the optimal solution is obtained.

## 4. Experiment

In order to verify the effectiveness of this method for patients, 12 groups of healthy volunteers were tested. All subjects gave their informed consent for inclusion before they participated in the study. The study was conducted in accordance with the Declaration of Helsinki, and the protocol was approved by the Ethics Committee of Yanshan University. Because the speed of physical expenditure for stroke patients is different in difficult tasks and due to the changes of the assistant force parameter K, maintaining the foot in a safe area requires a different level of initiative. With the increase of the difficulty coefficient, the K value of the assisted force parameter decreases gradually. Meanwhile, the patients’ goal participation will increase during the training process, which may lead to a rapid decline in the patients’ physical strength. So the human–computer interaction exerted by the patient at the end of the robotic chain is related to the degree to which the patient participates in the task. The degree of the patients’ participation in tasks is also closely related to the difficulty of the tasks. To verify this point, the force/moment of three joints is calculated as the terminal force in robot coordinates. Secondly, the obtained data yields an observation of the changes of the calculated end force in the training cycle and a comparison of the position of the end of the robot under different parameter K values of the assisted force.

**Figure 8:**
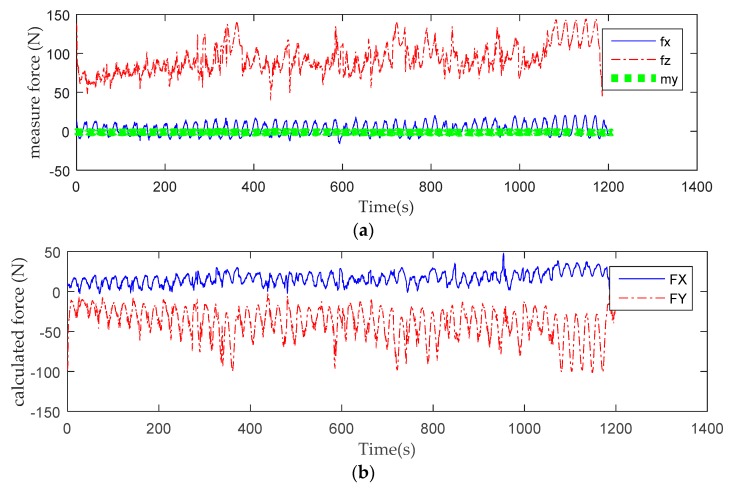
Sensors measuring terminal force during training: (**a**) Human–computer interaction of six-dimensional force acquisition under the training task of assistant force parameter K = 0.4; (**b**) the calculated terminal force in robot coordinates under the training task of assistant force parameter K = 0.4.

**Figure 9:**
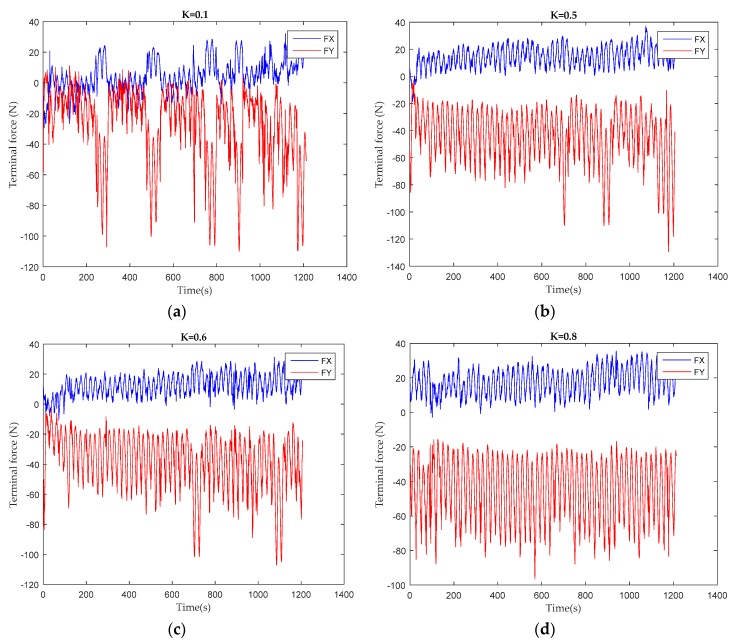
Terminal force during different difficulty tasks training: (**a**) the training task of assistant force parameter K = 0.1; (**b**) the training task of assistant force parameter K = 0.5; (**c**) the training task of assistant force parameter K = 0.6; (**d**) the training task of assistant force parameter K = 0.8.

Figure 8 shows the end force after transforming the data collected by the sensor into the end force of the robot coordinate after eliminating the self-weight of the robot. Under the assists force with K = 0.4 task difficulty, the human–robot interaction force keeps at a relatively low level for a period of time at the beginning of training. At 380 s, the volunteer is too weak to bear his own weight to complete the task, and the Human–robot interaction has reached its first peak. At 400 s, the volunteer challenges himself again and strives to achieve the goal of the task, so the human–robot interaction force declines rapidly because the volunteer takes the initiative to bear the weight of their limbs. However, the second phase of maintaining a lower level is shorter than the first phase, and the second peak of the interaction force appears. At the end of the training, it is difficult for the volunteer to bear part of the body weight again in order to achieve the goal of the task, so the interaction force, which is almost entirely composed of the volunteer’s limb weight, keeps at a high level. The variations in the time period of repeated challenges for a volunteer at different task difficulties are shown in Figure 9.

The picture above is the terminal force of the first volunteer in training tasks with different K values of the assist force. After a questionnaire survey of the volunteer, the K = 0.1 of assist force task for the patient is “over–challenge”, and the K = 0.5, K = 0.6 of the assist force task are “challenge”, with the K = 0.8 of the assist force task being “under-challenge”. Under the over-challenge task, there being heavier limb weights to load, the volunteer’s physical exertion is fast and there appear many peaks in the human–robot interaction force. With the increasing participation of assistive forces in training, volunteers need less initiative to achieve task goals. This phenomenon can be clearly seen by observing the relationship between the end position and the safe passage.

**Figure 10:**
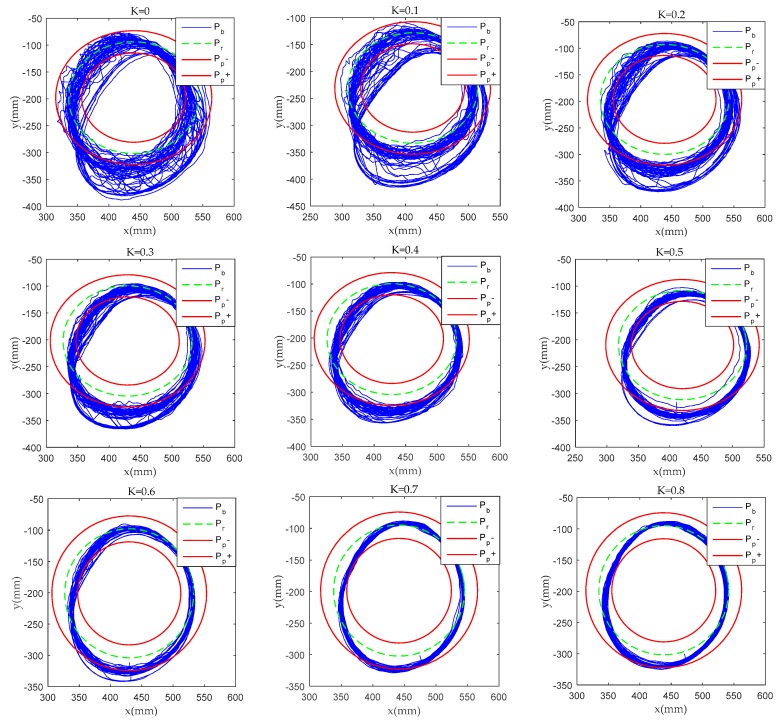
Terminal position during different difficulty tasks training.

Figure 10 shows the relationship between the terminal position of the robot and the safe passage of the target in the different difficulty training tasks for the first volunteer. The blue line is the rehabilitation robot terminal position, and the green line is the target terminal trajectory, while the red line is the safe passage. The difficulty of K = 0, K = 0.1, K = 0.2, K = 0.3 assist force tasks are “over-challenge”, K = 0.4, K = 0.5, K = 0.6 assist force tasks are “challenge”, and K = 0.7, K = 0.8 assist force tasks are “under-challenge”. It is difficult for the volunteer to complete the task goal in the over-challenged task. In the under-challenged task, it’s easy for volunteers to reach the goal position.

The degree of patient participation under different task difficulties is reflected in the fluctuation of human–machine interaction mechanical signals, and the feature fluctuation represents the fluctuation of signal data in this period. The greater the fluctuation of the feature, the greater the degree of dispersion, as it is more sensitive to signal fluctuation, and it is more suitable to be used as an input parameter of the detection model for patient participation. The sample data is processed by using four indicators, describing the degree of dispersion of the signals, the interquartile range, and the variance. The above features in the data are statistically analyzed to judge their significance and correlation under different volunteer states. Significance analysis is undertaken to compare the feature data of “under-challenge” and “over-challenge” volunteer states with that of the “challenge” state, in order to judge that the feature data have significant differences in the three states. The correlation is done to compare the insignificant features with the degree of volunteer participation, whether and if there is correlation. This feature will still be used as an input parameter to train the support vector machine. This paper extracted the preliminary feature variables in Table 1.

Ten volunteers were selected to carry out the experimental verification of the participation and task difficulty detection. Each volunteer was trained in 10 difficult tasks that lasted from 15 min to 20 min. In order to eliminate the influence of physical energy consumption between each experiment, they were conducted one day apart. After the end of the experiment, a questionnaire survey was conducted on the difficulty of the task, which is divided into three participation levels: “under-challenge”, “challenge”, and “over-challenge”. As part of the experiment, the data of hip and knee joint torque, plantar six-dimensional force, and terminal trajectory were collected. With the different participation of assistive force, there are different performances of the terminal force and position. The characteristic quantities were extracted, as shown in Table 2, from the training data. The training data characteristic quantities of volunteers were then compared to their classification, according to the predicted task difficulty. The pairwise t-test comparisons of the characteristic quantities were statistically analyzed to verify whether the characteristic quantities are significantly different under different task difficulties. Comparisons of the characteristics of each two difficult tasks, using one-way repeated measure ANOVA, were done separately. Table 2 shows the results of the significance analysis of the characteristic quantity in the difficulty of the three tasks. *p* value is the test probability, F value is the effect of random error. When *p* value is less than 0.05, the characteristic quantity has significant difference under different difficult tasks.

**Figure 11:**
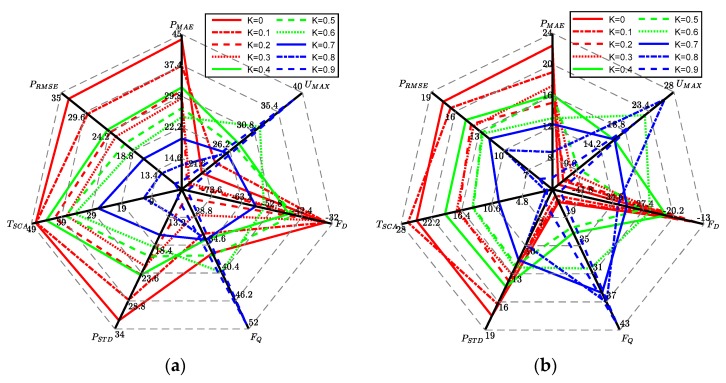
Characteristic quantity of training data under different task difficulties: (**a**) characteristic quantity of Volunteer 1^#^ training data; (**b**) characteristic quantity of Volunteer 2^#^ training data.

The significance analysis of the characteristic quantity from the training data of 10 volunteers shows that there are obvious differences when comparing the ***P*_RMSE_**, ***P*****_STD_**, ***T*_SCA_**, ***P*_MAE_** among different volunteers. The *p* value of ***F*_Q_**, ***U*_MAX_**, ***F*****_D_** are greater than 0.05, only in the case of the difficult and medium groups. For ***F*_Q_**, ***U*_MAX_**, ***F*****_D_** there are obvious differences in other groups, as it can distinguish the difficulty of the under-challenge tasks. Although ***P*_OR_** has significant differences, its value is rough and its stability is not high. Accordingly, ***P*_RMSE_**, ***P*****_STD_**, ***T*_SCA_**, ***F*_Q_**, ***U*_MAX_**, ***F*****_D_**, ***P*****_MAE_** are used as feature inputs for volunteer participation and task difficulty classification. Figure 11 shows more intuitively the difference in training characteristics among three difficulty levels for each volunteer.

Figure 11 shows the characteristics of two volunteers under different task difficulty. Among them, the red line is under the task difficulty of over-challenging and difficult; the green line is under the task difficulty of challenging; the blue line is under the task difficulty of under-challenge. ***P*_RMSE_**, ***P*****_STD_**, ***T*_SCA_**, ***F*****_D_**, ***P*****_MAE_** are positively correlated with the task difficulty evaluation, and ***F*_Q_**, ***U*_MAX_** are negatively correlated with the task difficulty evaluation.

The training data of 100 groups of 10 volunteers receiving the test were used as training sample data. At the same time, another two volunteers were randomly selected as the predictive group. Two volunteers in the predictive group were trained in all tasks with different difficulty levels, and questionnaires were conducted on task difficulty. Their training data is used as predictive sample data. Extracted feature quantities ***X_s_*** as input from 100 sets of experimental sample data and the training set known category information ***Y_s_*** (Task Difficulty of Patient Evaluation) were taken as output. The prediction model based on QPSO-MLSSVM hybrid optimization algorithm and the two comparison models based on MLSSVM algorithm and a neural network algorithm were established. QPSO is an iterative optimization that optimizes the parameters ***C*** and ***σ*** in the MLSSVM algorithm to improve the generalization ability and prediction accuracy of the model. The 20 sets of data in the test set are similar to the ones in the training set, and seven feature quantities ***X_l_*** are extracted as inputs of the existing model, and the predicted output ***Y_p_*** is obtained by the model operation. The accuracy of the model was evaluated by a minimum mean square deviation operation between ***Y_l_*** (Task Difficulty of Patient Evaluation) and ***Y_p_***.

In the analysis of the results, the training samples obtained from the mathematical model cannot directly reflect the prediction ability of the model. Further model evaluation can be achieved by comparing the prediction data of the training samples with the real data. Common evaluation indexes of the model include MSE, RMSE, correlation coefficient, and so forth. In this paper, the mean square error and the correlation coefficient are used to evaluate the model.

**Figure 12:**
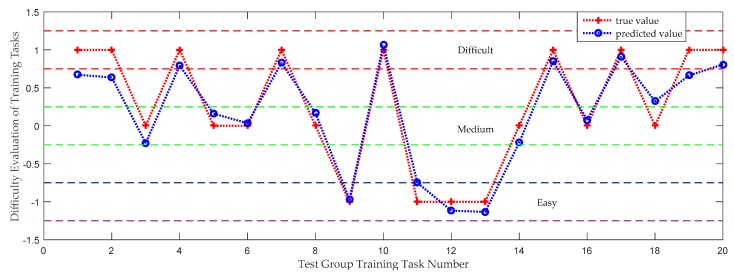
Comparison between task difficulty prediction and reality of test group.

The data from 100 datapoints of the volunteers’ participation status were input into the prediction model to train the model, and the prediction tested on 20 datapoints. Correlation analysis was conducted on the actual values and predicted values of the data, and the linear fitting results are shown in Figure 12.

In order to further analyze the classification effect of the QPSO-MLSSVM support vector machine on the state of volunteer participation, the minimum mean square error (MSE) and mean absolute error (MAE) as well as Standard Deviation (MAPE) of the predicted and true values of various volunteer participation states was obtained, as shown in Table 3

Training concentration simply divides task difficulty evaluation into −1, 0, and 1. Because patients have different evaluation criteria for difficulty, the dynamic trend in training data is different, and the results after algorithm testing will be distributed around three values. If the test results are graded according to the difficulty of (−1.5, −0.75), (−0.25, 0.25), (0.75, 1.25), the accuracy of the test can reach 100%. In order to eliminate the result of slightly larger offset, the determination range is reduced to half of the original one that are (−1.25, −0.5), (−0.5, 0.5), (0.5, 1.5). And the test accuracy can still reach 80%. The matching result of task difficulty evaluation shows that the predicted value of task difficulty is close to the real value, which also verifies that volunteers’ evaluation of the difficulty of training tasks can be obtained from training data.

## 5. Discussion

Early rehabilitation training for stroke patients is very important and effective. While the rehabilitation robot can be used in the later stages of recuperation and even as a workout enhancer, the research work is aimed at the early stages of post-trauma rehabilitation. The aim is to re-train the nerve control and brain–body associations for typical movements of the affected limb. Overall, the success of training is measured in how quickly and effectively a patient regains normal control of their limbs. In order to make patients take the initiative to participate fully, while not letting the patient’s physical strength drop rapidly, dispelling the enthusiasm of patient training, is a complex problem. It is very important to choose the appropriate training task difficulty for patients. Therefore, this paper determines whether the current task difficulty is suitable for patient training to achieve optimal training effect based on the data of the patient in the training task. The final experiment in the paper proves that the matching degree of task difficulty evaluation of the two volunteers in the test group was worse than that of the test difficulty evaluation of the volunteers in the experimental group from the fitness curve. This is due to the volunteer’s subjective persistence and subjective evaluation of the difficulty of the task. However, the support vector machine task difficulty judgment model still has a prediction accuracy of 80% for the volunteer task difficulty evaluation of the test group. As the training data continues to increase and a variety of training information is introduced, the prediction accuracy of the judgment model will become higher and higher.

As can be seen from Figure 8 and Figure 10, when volunteers perform multiple training tasks with different difficulty, with the decrease of difficulty and the increase of the proportion of assistant power, the strength needed by volunteers to achieve the goal task position and the speed of physical consumption will be reduced. During clinical trials, most volunteers have emotional issues when performing challenging tasks. Most of them have low mood, and some are irritated. They need to continue their psychological counselling and speech encouragement to support their task training. When the volunteers were under challenging tasks, most of them felt bored and emotionally stable. It may have an effect on the experimental results for the frequency of speech encouragement during training. In this clinical trial, speech encouragement was given four times in each training process to minimize the influence of this factor. In the future, the research team will use a variety of measurements to study the emotional and physical characteristics of volunteers to verify their impact on rehabilitation training.

In this paper, the performance of volunteers in training tasks at different levels of difficulty is investigated in order to determine whether the task difficulty is appropriate and to verify and judge the past data. But it also proves the validity and universality of the assistant training strategy. This control strategy can maximize the ability of patients to actively participate in training. In the future, the research team will continue to study and improve upon such clinical trial data. It is expected that the task difficulty can be judged and predicted online, and then the assistant force can be adjusted in real time, so that patients can participate in training actively and optimally.

## 6. Conclusions

This paper studies a seated and reclining training lower limb rehabilitation robot with a multi-joint sensing system. In order to make the patient participate actively in the training task, an assistive force training control strategy and corresponding task difficulty are proposed. The multi-joint mechanical sensing system is used to solve the more accurate end mechanical model, and then the human–computer interaction force is detected. Clinical trials of 10 volunteers were conducted, and each volunteer underwent nine levels of difficulty training. Through the optimized support vector machines algorithm, quantitative features in the training data are taken as the input set, and the volunteer’s evaluation of the task difficulty is taken as the output set, and a task difficulty judgment model based on the volunteer training data is obtained. The training difficulty of two other volunteers, not in the original 10 persons training set, was predicted. It was verified that the difficulty judgment model of the task was universal and could exclude the influence of body size and weight. By comparing the prediction results of various algorithm models, the accuracy and convergence speed of the optimization algorithm are verified.

Future work will concentrate on extending the research to alternative models, such as described in the introduction, with a detailed comparison providing possible improvements to the data pipeline. The application will also benefit from a continuous expansion of the dataset, as more patient trials become available. This will also lead to the training data being judged and predicted online, and the difficulty of the task being adjusted in real time to optimize the rehabilitation effect of the patient in the future. As discussed throughout the paper, the patient’s perception of the difficulty of the training exercise influences their mood, behavior, and performance. As such, matching the patient’s perception is an important task in itself, even if the mechanical ground truth may be misrepresented. The desired end result for the rehabilitation robot, including future research, is a real-time online assessment which includes individual patient profiles, which should make patient subjectivity less relevant.

## Figures and Tables

**Table 1:** Characteristic parameters of volunteer participation.

Type	Description
*P*_RMSE_	Mean square error of position
*P*_STD_	Position standard deviation
*T*_SCA_	The proportion of time outside the safe passage
*F*_Q_	Inter-quartile range of terminal force
*U*_MAX_	Maximum value in frequency domain of terminal force
*f*_MAX_	Peak frequency in frequency domain of terminal force
*F*_D_	Component at frequency 0 in frequency domain of terminal force
*F*_VAR_	Variance of terminal force
*P*_OR_	Offset range of position
*Uh*_MAX_	Maximum value in frequency domain of volunteer motivation
*fh*_MAX_	Peak frequency in frequency domain of volunteer motivation
*P*_MAE_	Mean absolute error of position

**Table 2:** Significance comparison of characteristic quantities.

**Comparison**	***P*** **_RMSE_**		***P*** **_STD_**		***T*** **_SCA_**	
	**P**	**F**	**P**	**F**	**P**	**F**
Difficult/Medium	3.19 × 10^−4^	1.09 × 10^−11^	1.5 × 10^−4^	3.76 × 10^−10^	1.44 × 10^−3^	5.9 × 10^−4^
Difficult /Easy	2.74 × 10^−7^	3.2 × 10^−14^	4.26 × 10^−8^	1.08 × 10^−12^	2.06 × 10^−11^	1.9 × 10^−4^
Medium/ Easy	8.08 × 10^−9^	0.1	1.26 × 10^−8^	0.112	6.13 × 10^−10^	0.634
**Comparison**	***F*** **_Q_**		***U*** **_MAX_**		***f*** **_MAX_**	
	**P**	**F**	**P**	**F**	**P**	**F**
Difficult/Medium	0.844	0.0904	0.136	0.07	0.39	0.382
Difficult /Easy	9.97 × 10^−3^	0.01	6.37 × 10^−4^	0.028	0.028	0.007
Medium/ Easy	4.7 × 10^−3^	0.339	0.0147	0.65	0.154	0.066
**Comparison**	***F*** **_D_**		***F*** **_VAR_**		***P*** **_OR_**	
	**P**	**F**	**P**	**F**	**P**	**F**
Difficult/Medium	0.735	2 × 10^−5^	0.255	0.001	7.59 × 10^−6^	0.0035
Difficult /Easy	0.033	0.221	0.961	0.072	3.07 × 10^−13^	5.66 × 10^−11^
Medium/ Easy	0.0013	0.002	0.193	0.212	2.922 × 10^−8^	1.78 × 10^−5^
**Comparison**	***Uh*** **_MAX_**		***fh*** **_MAX_**		***P*** **_MAE_**	
	**P**	**F**	**P**	**F**	**P**	**F**
Difficult/Medium	0.0013	0.009	0.001	0.6789	0.0005	5.38 × 10^−12^
Difficult /Easy	0.1478	0.003	0.072	0.5531	3.19 × 10^−7^	4.29 × 10^−13^
Medium/ Easy	0.5216	6.06 × 10^−7^	0.929	0.8565	2.32 × 10^−9^	0.339

**Table 3:** Significant comparison of characteristic quantities.

	MSE	MAE	STD
Matching degree	0.0428	0.1822	0.1006
